# Changes in the Mucus Barrier during Cisplatin-Induced Intestinal Mucositis in Rats

**DOI:** 10.1155/2013/276186

**Published:** 2013-12-23

**Authors:** Hajime Yamamoto, Kazuhiko Ishihara, Yuko Takeda, Wasaburo Koizumi, Takafumi Ichikawa

**Affiliations:** ^1^Department of Internal Medicine, Kitasato University School of Medicine, Sagamihara, Japan; ^2^Department of Regulation Biochemistry, Kitasato University Graduate School of Medical Sciences, Kitasato University, 1-15-1 Kitasato, Minami-ku, Sagamihara, Kanagawa 252-0373, Japan

## Abstract

*Aim*. Gastrointestinal mucositis is a frequent complication of antineoplastic chemotherapy, but the effects of chemotherapy on mucosal defense mechanisms remain poorly understood. We studied the effects of cisplatin on mucin, one of the principal defense factors of the gastrointestinal mucosa, and evaluated the efficacy of two different types of H_2_-receptor antagonists against cisplatin-induced mucositis. *Methods*. Cisplatin (6 mg/kg) was administered intravenously to rats (day 0). The rats were sacrificed 1, 3, 7, and 11 days after treatment, and their stomach, jejunum, ileum, and colon were removed. Immunoreactivity of the mucosa was compared with the use of anti-mucin monoclonal antibody. To evaluate the efficacy of H_2_-receptor antagonists, either famotidine (3 mg/kg) or lafutidine (30 mg/kg) was given orally once daily on days 0, 1, and 2. Histological and biochemical findings were compared among the groups to assess effects on cisplatin-induced injury. *Results*. Cisplatin significantly altered the immunoreactivity and content of mucin in the small intestinal mucosa, especially in the ileum. Lafutidine protected against cisplatin-induced mucosal injury and attenuated decreased mucin accumulation. *Conclusion*. Cisplatin appears to alter the mucus barrier function in the intestinal mucosa. Lafutidine might effectively prevent chemotherapy-induced mucositis by activating intestinal mucus cells.

## 1. Introduction

Gastrointestinal (GI) mucositis, a frequent complication of antineoplastic chemotherapy, can reduce treatment effectiveness because it leads to dose reductions, increased healthcare costs, and an impaired quality of life [1, 2]. Clinical practice guidelines recommend antiacid secretory drugs such as H_2_-receptor antagonists for the prevention and treatment of GI mucositis [3–6]. Recently, some of the newer H_2_-receptor antagonists (so-called second-generation H_2_-receptor antagonists) have been frequently used in Japan. These agents have a unique component structurally differing from conventional H_2_-receptor antagonists and promote gastric mucosal defense mechanisms, including mucus secretion [7]. Although medical therapy has improved the management of symptoms in patients with chemotherapy-induced mucositis [3–6], their effects on mucosal defense mechanisms remain poorly understood.

At present, 5-fluorouracil (5-FU) and cisplatin-based chemotherapy is most widely used to treat advanced GI cancer [8, 9]. Several experimental studies have investigated the mechanisms of small-intestinal-mucosal injury induced by 5-FU [10, 11], and we have recently reported significant changes in the mucus barrier of the rat during 5-FU-induced GI mucositis [12]. On the other hand, cisplatin is well known to be associated with renal toxicity [13, 14], but there is a dearth of information about its effects on the GI mucosa.

Mucin, a major component of mucus, is considered to play an important role in the physiological defense of the GI mucosa. Our previous studies showed quantitative and qualitative changes in GI mucin in normal and diseased animals, as well as in humans, and demonstrated the importance of mucin in the GI mucosal barrier [15–18]. We have also established several monoclonal antibodies (mAbs) that react with mucin synthesized and secreted by specific mucus-producing cells of the rat GI mucosa [18–20].

The first objective of this study was to sequentially compare the effects of cisplatin on mucus in different portions of the rat GI tract. Next, we evaluated the efficacy of two different types of H_2_-receptor antagonists, famotidine, and lafutidine, against cisplatin-induced intestinal-mucosal injury in rats. We also assessed the effects of these drugs on ileal mucin accumulation. Famotidine is a well-known conventional H_2_-receptor antagonist, and lafutidine is a second-generation H_2_-receptor antagonist group, characterized by possessing a six-membered aromatic ring [7].

## 2. Material and Methods

### 2.1. Animals and Drug Treatment

Seven- or eight-week-old male Wistar rats (CLEA-Japan, Tokyo, Japan) were used in this study. The animals were housed in our animal care facility for 1 to 2 weeks to allow body weight to stabilize. At the beginning of the experiment, the animals were weighed. During treatment, food and water were provided *ad libitum*. The animals were weighed again and sacrificed on the assigned day of each experiment. The stomach, proximal and distal small intestine (corresponding to the jejunum and ileum, resp.), and colon were removed. This study was conducted in accordance with the guidelines of the Animal Laboratory Center of Kitasato University School of Medicine.

### 2.2. Effects of Cisplatin on Body Weight and GI Tract

Rats were divided into a cisplatin group and control group (*n* = 4-5 per group). In the cisplatin group, cisplatin (Sigma-Aldrich Inc., St. Louis, MO, USA) was suspended in saline solution and injected into a tail vein at a dose of 6 mg/kg on day 0. In the control group, rats were similarly given a single dose of saline solution on day 0. Body weight, histological changes of the stomach, jejunum, ileum, and colon, and the mucin content of these organs were assessed on days 1, 3, 7, and 11 days after injection as described below.

### 2.3. Effects of H_**2**_-Receptor Antagonists on Cisplatin-Induced Mucosal Damage

Rats were divided into the following 4 groups (*n* = 4-5): a control group, cisplatin group, cisplatin plus famotidine group, and a cisplatin plus lafutidine group. Cisplatin was suspended in saline solution and injected into a tail vein at a dose of 6 mg/kg on day 0 in the cisplatin group, cisplatin plus famotidine group, and the cisplatin plus lafutidine group. The control group similarly received saline solution on day 0. In the cisplatin plus famotidine group and the cisplatin plus lafutidine group, the respective antiulcer drugs were suspended in 0.5% carboxymethyl cellulose (CMC) (Kanto Chemical Co. Inc., Tokyo, Japan) solution immediately before use. The first dose of each antiulcer drug (famotidine 3 mg/kg; lafutidine 30 mg/kg) was given by oral gavage 30 minutes before the injection of cisplatin on day 0. Additional doses of famotidine or lafutidine were similarly given once daily on days 1 and 2. Control animals received 0.5% CMC instead of the antiulcer drugs. Rats in all groups were fasted from day 2 onward and were sacrificed on day 3.

### 2.4. Histological Examination

Specimens of each tissue were immediately fixed for 3 h in Carnoy's solution, freshly prepared as described elsewhere [21]. After fixation, the tissues were dehydrated in ethanol, cleared in xylene, embedded in paraffin, and sliced into 3 mm thick paraffin sections, which were then prepared for immunostaining with antimucin monoclonal antibodies (mAb). Immunohistochemical staining was done using the avidin-biotin-peroxidase method and an LSAB2 Kit (Dako, Carpinteria, CA, USA). Briefly, endogenous peroxidase activity was blocked with 0.3% H_2_O_2_, and the tissue was then sequentially incubated with 10% (v/v) normal swine serum, an anti-mucin mAb (PGM34), biotinylated anti-mouse immunoglobulins, streptavidin horseradish peroxidase (HRP), and 0.02% 3,3-diaminobenzidine in 50 mM Tris-HCl, pH 7.6, containing 0.005% H_2_O_2_. Counterstaining was done with hematoxylin and eosin (H-E). The immunohistochemical reactivity of the mAb was assessed with the use of an optical microscope. Villus height in the epithelium of the jejunum and ileum was measured in 5 rats per group. The villus height was measured at 3 sites of 3 high-power fields (total, 9 sites) in each rat and the mean value and standard deviation were calculated.

The epitope of the mAb PGM34 was recently shown to be a specific sulfated oligosaccharide of the mucin molecule. This mAb stains all goblet cells of rat small intestine [20]. The Ki-67 protein is present during all active phases of the cell cycle (G_1_, S, G_2_, and mitosis) but is absent in resting cells (G_0_), making it an excellent marker for determining the so-called “growth fraction” of a given cell population [22–24]. Paraffin sections of the small intestinal mucosa, 3 *µ*m thick, were used for immunostaining with Ki-67, performed by the same method as outlined above. A primary mAb against Ki-67 (MIB-5; Dako, Glostrup, Denmark) was used instead of the antimucin mAb. The number of Ki-67 positive cells was measured for each sample.

### 2.5. Biochemical Examination

Specimens from each tissue were lyophilized and powdered for extraction of mucin as described previously [15]. Each sample was suspended in 50 mM Tris-HCl, pH 7.2, containing 2% Triton X-100 (Triton-Tris buffer), homogenized, and then incubated at 37°C for 1 h. After centrifugation at 8000 g for 30 min at 4°C, the supernatant was collected, and an aliquot was applied to a Bio-Gel A-1.5 m column and eluted with the Triton-Tris buffer. The void volume fraction (Fr-1) monitored by hexose measurement was collected as mucin. The hexose content of this fraction was measured by the phenol-sulfuric acid method using galactose as the standard. The mucin content (Fr-1 hexose value) was expressed as micrograms of Fr-1 hexose per gram of dry tissue weight.

### 2.6. Statistical Analysis

Differences in mean values among groups were analyzed by one-way analysis of variance with Scheffe's test; *P* values of less than 0.05 were considered to indicate statistical significance.

## 3. Results

### 3.1. Body Weight Change

**Figure 1:**
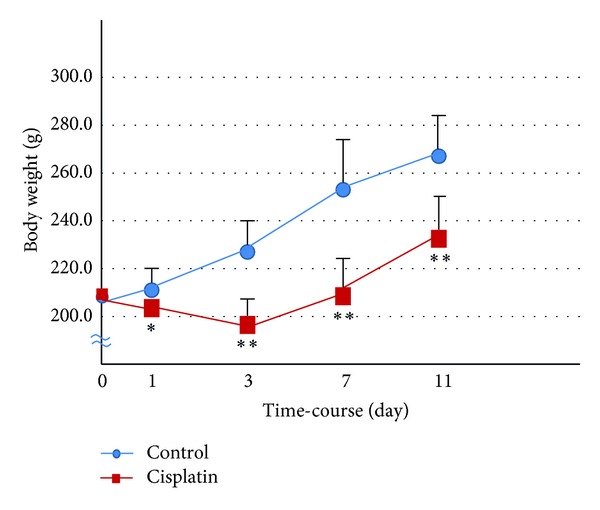
Time-course of body weight of rats on 1, 3, 7, and 11 days after treatment with cisplatin. The body weight of each rat was measured immediately before sacrifice. Data are presented as means ± SE (*n* = 4-5). *P < 0.05 and **P < 0.01.

During the 11-day study period, body weight increased in a stepwise fashion in the control rats, but body weight gain significantly decreased after the injection of cisplatin (Figure 1). During the first 3 days after treatment, body weight decreased in the rats given cisplatin (6 mg/kg i.v.). As shown in Table 1, there was virtually no change in the body weight of rats given cisplatin plus famotidine as compared with those given cisplatin alone. In contrast, lafutidine inhibited cisplatin-induced body weight loss.

### 3.2. Changes in Morphology and Mucin Content of GI Mucosa after Cisplatin Treatment

**Figure 2:**
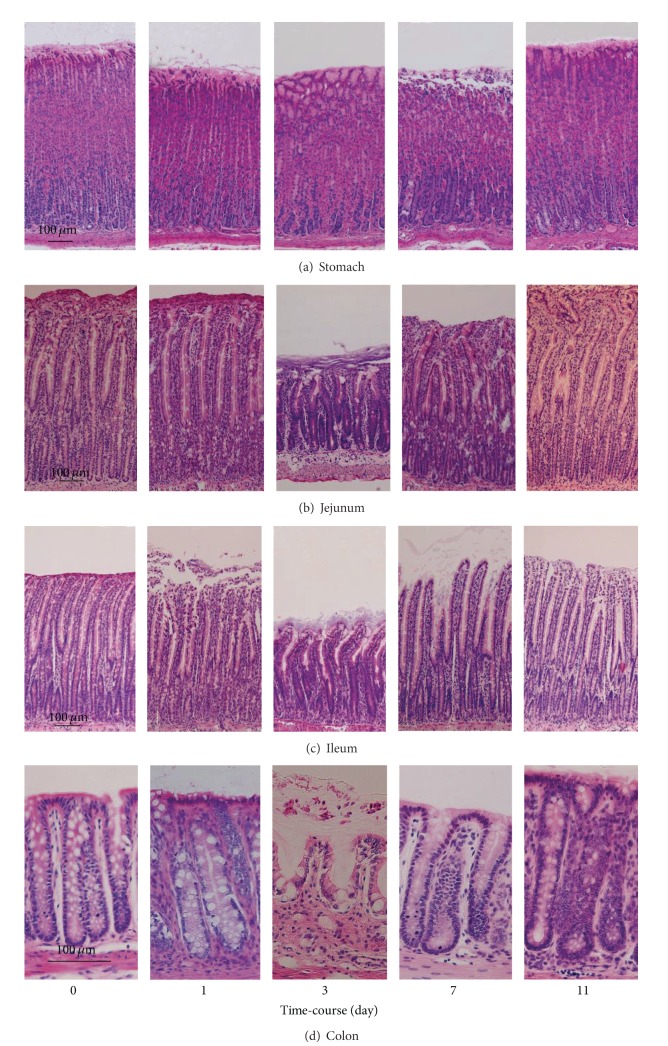
Microscopical findings of the gastrointestinal mucosa stained with hematoxylin and eosin. The animals were given cisplatin 6 mg/kg i.v. and were sacrificed at various time points (1, 3, 7, and 11 days) after treatment.

**Figure 3:**
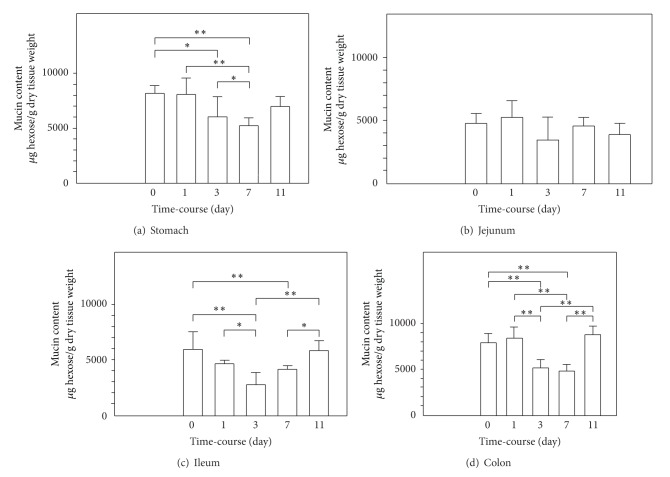
Time-course of the mucin content of the gastrointestinal mucosa. The animals were given cisplatin 6 mg/kg i.v. and were sacrificed at various time points (1, 3, 7, and 11 days) after treatment. Mucin content is expressed as micrograms of Fr-1 hexose per gram of dry tissue weight for each type of mucosa. Data are presented as means ± SE (*n* = 4-5). *P < 0.05 and **P < 0.01.

Mucosal damage characterized by epithelial sloughing and mucosal ulceration of villous tips was detected in the GI tract mucosa of each rat after injection of cisplatin. On day 3 after treatment with cisplatin, severely injured epithelial mucosa was seen in the small intestine, especially the ileum, whereas evidence of GI mucosal injury was minimal on day 1. As shown in Figures 2(b) and 2(c), cisplatin treatment markedly decreased the villus height in the intestine. The villus area fully recovered by day 11 after cisplatin challenge. The simultaneously measured mucin contents of the rat GI mucosa are shown in Figure 3. The content was most markedly reduced in the ileum on day 3 and increased thereafter. On day 11 after cisplatin challenge, the ileal mucin content had returned to the baseline level.

### 3.3. Effects of H_**2**_-Receptor Antagonists on Cisplatin-Induced Mucosal Damage

**Figure 4:**
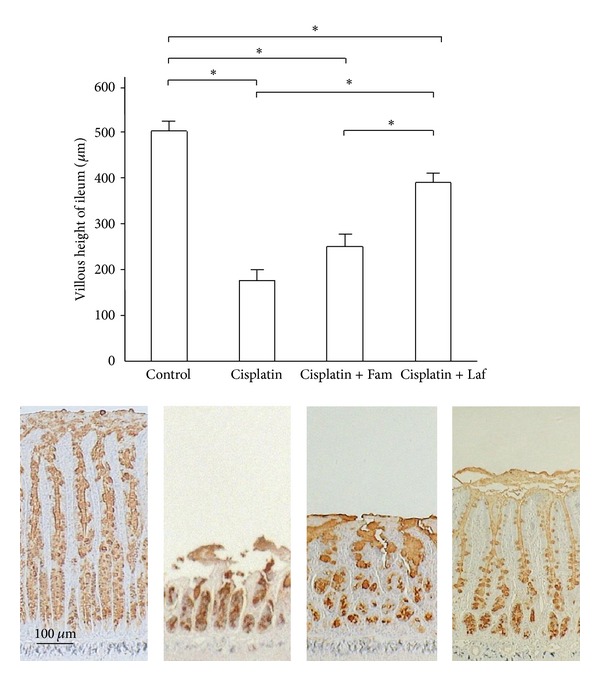
Comparison of the effects of H_2_-receptor antagonists on cisplatin-induced ileal mucosal damage as evaluated by immunostaining with PGM34. The animals were given cisplatin 6 mg/kg i.v. and were sacrificed 3 days after treatment. The villus height of the ileum significantly decreased in the cisplatin group as compared with the control group and significantly increased in the cisplatin + Laf group as compared with the cisplatin group. Means (±SE) *n* = 5 (each group); Fam: famotidine; Laf: lafutidine; *P < 0.05.

The effects of two types of H_2_-receptor antagonists on cisplatin-induced damage of the ileal mucosa were compared on day 3 after cisplatin injection. As shown in Figure 4, in the control rats, immunohistochemical reactivity for PGM34 was detected in goblet cells, as well as in the surface mucus gel layer of the ileum. Cisplatin treatment markedly reduced the villus height and decreased the number of PGM34-positive goblet cells. In the rats treated with cisplatin plus lafutidine, appreciable damage was rarely found in sections of the ileal mucosa, whereas famotidine did not prevent cisplatin-induced ileal mucosal damage. Likewise, Table 2 compares the effects of the H_2_-receptor antagonists on the mucin content of the ileal mucosa on day 3 after the induction of mucosal damage by cisplatin. The mucin content of the ileum decreased after treatment with cisplatin to 67.4% of the control value. Lafutidine significantly inhibited the cisplatin-induced decrease in the ileal mucin content to 86.9% of the control value. In contrast, concurrent treatment with famotidine had no discernible effect on the mucin content as compared with treatment with cisplatin alone.

**Figure 5:**
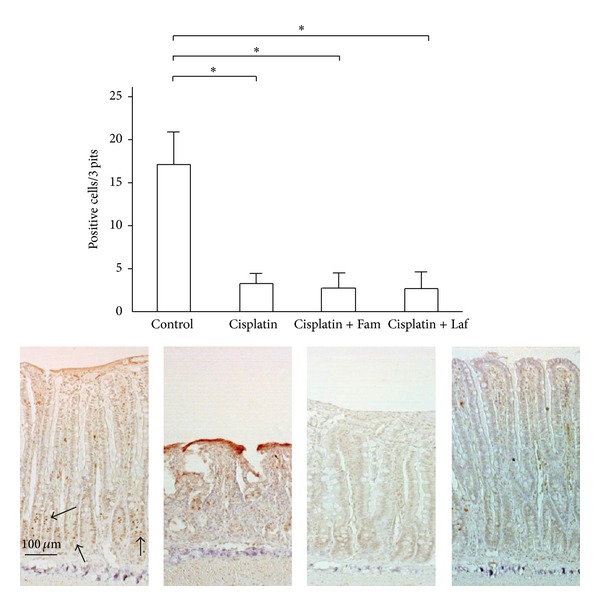
Changes in the immunoreactivity of Ki-67 of the ileal mucosa after treatment. The number of Ki-67-positive cells in three pits is shown. The arrows indicate Ki-67-positive cells. Means (±SE) *n* = 5 (each group); Fam: famotidine; Laf: lafutidine; *P < 0.05.

Figure 5 shows the morphologic changes of ileal Ki-67-positive cells after treatment. In the control rats, immunohistochemical reactivity for Ki-67-positive cells was detected in the proliferative zone. Cisplatin treatment remarkably reduced the number of Ki-67-positive cells. In the animals treated with cisplatin plus lafutidine, the expression of Ki-67-positive cells decreased. Nonetheless, the ileal mucosa was maintained in the cisplatin plus lafutidine group.

## 4. Discussion

We found that intravenous injection of cisplatin in a single dose of 6 mg/kg caused GI mucosal damage altered the GI mucin content and inhibited body weight gain of rats. Our results are consistent with those of prior studies showing that mucosal damage characterized by epithelial sloughing of villous tips occurs 3 to 7 days after treatment with cisplatin [25]. In our preliminary study, 10 mg/kg of cisplatin was also found to induce GI mucosal injury in rats, similar to 6 mg/kg, but a considerable number of animals died after treatment with the higher dose. Previous works showed that 3 mg/kg of cisplatin-induced nephrotoxicity, but not GI mucosal injury in rodents [26]. Taken together, these findings indicate that the dose of cisplatin used in the present study was appropriate for evaluating effects on the GI mucosa. The present data demonstrate that cisplatin, at clinically appropriate doses, not only inhibits renal function but also influences mucin metabolism.

Accumulation of mucin in the GI mucosa is closely related to mucosal protective capability, acting as a mucus barrier [15–17]. In the stomach, mucin is a key element in protecting the gastric epithelium against various irritants [15, 17]. The present study showed that a decreased mucin content in all parts of the GI tract is a cause of mucositis after treatment with cisplatin. Our most notable finding was a remarkable cisplatin-induced reduction in the mucin content of the ileum. Although the protective property of intestinal mucin has received limited attention as compared with gastric mucin, our results suggest that the ileal mucosa is especially vulnerable to the adverse effects of cisplatin.

A specific type of mucin is expressed in distinct mucus-producing cells of the mammalian GI tract [21, 27]. Using the original antimucin mAb PGM34, we studied the preventive effect of lafutidine on cisplatin-induced alterations in rat ileal mucus. This mAb recognizes the sulfuric acid residue structure attached to mucin molecules [20]. Our results showed that lafutidine prevented cisplatin-induced small intestinal mucosal damage in rats. Lafutidine, a second-generation H_2_-receptor antagonist, has been reported to stimulate mucin accumulation independently of its H_2_-receptor antagonistic properties and to protect against necrotizing-agent-induced mucosal damage in the rat [7]. Moreover, at clinical dose levels, lafutidine not only inhibits acid secretion but also strengthens the mucus barrier of the human gastric mucosa [28]. Our finding that lafutidine prevented mucosal damage indicates that changes in the mucus barrier are the “causes” of cisplatin-induced mucositis. Although further studies are needed to clarify the functions of specific types of mucin, a reduction in PGM34-positive mucin may contribute to the initiation or progression (or both) of chemotherapy-induced mucosal injury in the rat small intestine.

The results of Ki-67 immunohistochemical staining proved that cisplatin alters cell proliferation in the rat intestinal epithelium. Consequently, attenuation of cisplatin-induced mucositis might be attributed to a reduction in its growth-inhibitory activity. Recently, supplementation of nutrients such as glutamine and vitamins was shown to attenuate cisplatin-induced mucosal damage by increasing intestinal-cell turnover [25, 29]. Our present study showed that lafutidine prevented cisplatin-induced alterations in rat intestinal mucus, without affecting cell turnover. Our previous study showed that lafutidine directly stimulated mucin production by rat mucus cells [7]. Thus, the preventive effect of lafutidine against cisplatin-induced intestinal damage may be attributed to the increased production of mucin by goblet cells that remained viable after cisplatin treatment.

In this study, famotidine did not attenuate the morphologic alterations or the changes in the mucin content of the intestinal mucosa in rats treated with cisplatin. First-generation H_2_-receptor antagonists such as cimetidine and famotidine have been reported to reduce the production and secretion of rat GI mucin [7]. Our findings suggest that famotidine did not promote the function of goblet mucus cells in this study. Although further investigations are needed to clarify the detailed mechanism of cisplatin-induced intestinal injury, the activation of the goblet cells, if appropriately manipulated, might lead to more effective prevention of cisplatin-induced GI mucositis.

In conclusion, our study had two major findings. First, alteration of the mucus barrier function is a cause of cisplatin-induced mucositis. Second, lafutidine might effectively prevent chemotherapy-induced mucositis by activating intestinal mucus cells.

## Figures and Tables

**Table 1:** Body weight of the rats before and 3 days after treatment in each experimental group.

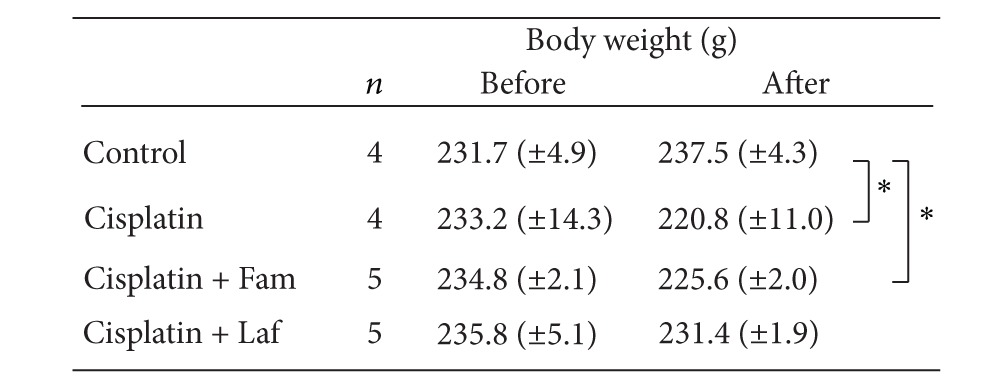

Fam: famotidine; Laf: lafutidine.

Means (±S.E), *P < 0.05.

**Table 2:** Changes in the mucin content of the ileal mucosa.

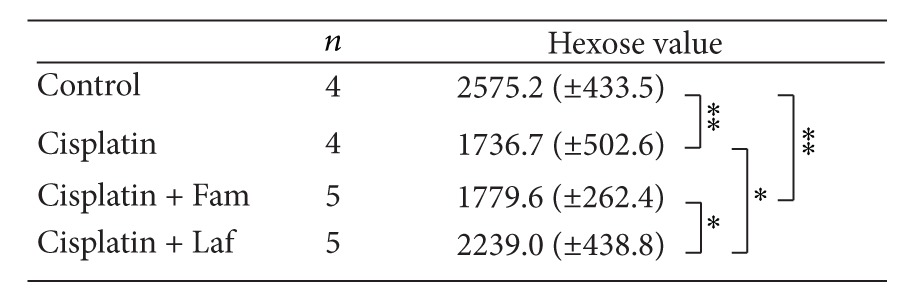

The animals were given cisplatin 6 mg/kg i.v. and were sacrificed 3 days after treatment.

Mucin content is expressed as micrograms of Fr-1 hexose per gram of dry tissue weight for each mucosa.

Fam: famotidine; Laf: lafutidine.

Means (±S.E), *P < 0.05, and **P < 0.01.
